# Colonoscopic screening for colorectal cancer improves quality of life measures: a population-based screening study

**DOI:** 10.1186/1477-7525-4-82

**Published:** 2006-10-18

**Authors:** Doug Taupin, Sharon L Chambers, Mike Corbett, Bruce Shadbolt

**Affiliations:** 1 Gastroenterology and Hepatology Unit and Centre for Advances in Epidemiology and Information Technology, The Canberra Hospital, Yamba Drive, Garran, Australian Capital Territory, Australia

## Abstract

**Background:**

Screening asymptomatic individuals for neoplasia can have adverse consequences on quality of life. Colon cancer screening is widespread but the quality of life (QOL) consequences are unknown. This study determined the impact of screening colonoscopy on QOL measures in asymptomatic average-risk participants.

**Methods:**

Asymptomatic male and female participants aged 55–74 years were randomly selected from the Australian Electoral Roll or six primary care physicians' databases. Participants completed the Short-Form (SF-36) Quality of Life Assessment at baseline and at a mean of 39 days after colonoscopy. Outcome measures were (i) significant changes in raw scores in any of the eight SF-36 domains assessed following colonoscopic screening and (ii) improvements or declines in previously validated categories, representing clinically significant changes, within any of the eight SF-36 domains.

**Results:**

Baseline QOL measures were similar to those of a matched general population sample. Role Limitations due to Emotions, Mental Health and Vitality raw scores significantly improved following colonoscopy (P < 0.05, 2-tailed t-test). Health ratings according to Category were similar (same clinical status) in the majority of participants. However, 30% participants recorded clinically significant improvement in the Mental Health and Vitality domains (P < 0.05, Wilcoxon Signed-Ranks test). This improvement was not offset by declines in other domains or in other participants. Improvement in QOL was not related to colonoscopy results.

**Conclusion:**

Average-risk persons benefit significantly from colon cancer screening with colonoscopy, improving in Mental Health and Vitality domains of Quality of Life. This improvement is not offset by declines in other domains.

## Background

Colorectal cancer is a common disease with a long lead-time and easily recognised precursor lesions, making screening a rational and effective means of prevention. Colonoscopy from age 50 is accepted as an accurate and cost-effective screening modality of colorectal cancer screening, but is not yet the 'preferred' strategy [1,2].

The act of screening asymptomatic individuals for cancer or precancerous lesions may result in health consequences, even in those not found to be screen-positive. Potential consequences include increased anxiety, reported among participants in screening for breast cancer, ovarian cancer, and prostate cancer [3-5]. However, while there is evidence that colorectal cancer screening may be the most effective form of cancer screening, there is little published data regarding either adverse or beneficial effects of screening on participants' health status. On the other hand, severe adverse effects of screening by colonoscopy, including haemorrhage and colonic perforation, are universally reported. While important, these indicators are relatively rare events, with major procedure-related morbidity in 0.3% [6]. Consequently, in an attempt to determine the health consequences of colonoscopic screening, we included a health-related quality of life measure before and after screening in a study of 231 participants recruited from the Australian Electoral Roll and primary care [7].

## Methods

### Criteria for selection of subjects

The study was originally designed to test the hypothesis that recruitment for colonoscopic screening from general practice would achieve better participation than recruitment from the electoral roll. The sample size was based on (i) the number of subjects aged 55–74 years needed to achieve 100 to 125 colonoscopies in each of two arms and (ii) the number of invitees to determine differences in recruitment [7]. Asymptomatic, male and female participants aged 55–74 years were randomly selected from the Australian Electoral Roll or six primary care physicians' databases and invited by letter to participate in colorectal cancer screening by colonoscopy. Participants were ineligible for inclusion if they had gastrointestinal symptoms requiring attendance at a primary care physician in the previous 12 months, colonoscopy or barium enema within the previous 10 years, significant co-morbidity (American Society of Anesthesiologists class III or greater), a prior diagnosis of cancer (not including non-melanomatous skin cancer), previous colonic surgery, or therapeutic anticoagulation.

Subjects attended the study site on three occasions. At Visit 1, an information sheet was provided and written consent for the study was obtained. We asked participants about their medical history, including medications, family history of cancer, and previous screening behaviour. Eligibility criteria were assessed. At Visit 2 colonoscopy was performed. All colonoscopy participants underwent a physical examination to record their fitness for sedation. Physician or nurse sedationists administered sedation with a combination of fentanyl, midazolam and propofol. At Visit 3, participants received results of the colonoscopy and results of histopathology where relevant. We sent a letter to the participant's primary care physicians, summarising the results and a providing a recommendation for surveillance.

The study was conducted according to the National Health & Medical Research Committee guidelines. The Australian Capital Territory Human Research Ethics Committee and the Calvary Hospital Medico-Moral Human Research and Ethics Committee approved the protocol (10 December 2001).

### Health assessment measures

Health-related quality of life was measured using the Short Form-36 (SF-36) instrument version 1.8–9 SF-36 questionnaires were completed by the participants at Visits 1 and Visit 3 with assistance from a Study Investigator. Adverse events were assessed at Visit 2 and Visit 3. At Visit 3, colonoscopy findings were discussed and a recommendation for repeat colonoscopy was made (five years for first-degree relative with colorectal cancer or finding of adenomatous polyp, ten years for normal findings and no first-degree relative with colorectal cancer).

### Statistical analysis

In addition to descriptive statistics, we carried out paired t-tests comparing SF-36 scores before and after colonoscopy. We performed ANOVA to examine the effect of potential confounding factors on changes in SF-36 scores. Potential confounding factors assessed were (i) finding of adenomatous polyps at colonoscopy; (ii) presence of adverse events, excluding failure to retrieve polyp; and (iii) participants' ratings (0–100 on a Visual Analog Scale) of discomfort and overall satisfaction with the procedure [7]. We performed unpaired t-tests comparing participants' SF-36 scores to those of respondents from an independent population survey *[Shadbolt B and Craft PS. Extending the use of the SF-36 in clinical practice. Health and Quality of Life Outcomes, Accepted for Publication]*.

In comparing the pre- and post-SF-36 scores we also employed 'clinically meaningful' cut points to interpret the distributions obtained. Briefly, SF-36 data from an Australian prospective cohort study of 5,668 hospital patients *[Shadbolt B and Craft PS. Extending the use of the SF-36 in clinical practice. Health and Quality of Life Outcomes, Accepted for Publication] *were used to determine Receiver-Operator curves in each SF-36 domain based on known health states in these patients. Cut points were applied to these Receiver-Operator curves to yield five categories (except the Role Physical domain, which has four validated categories). These categories were retested against raw data to confirm levels of sensitivity and specificity to 'clinically poor' health and 'clinically good' health. In the present study, movement by subjects between these categories before and after colonoscopy were used to determine whether or not a participant had a clinically worse or better SF-36 scale score after colonoscopy. Statistical analysis was performed with SAS 9.1 (SAS Institute, Cary NC). Incomplete data – data missing for one or more domains of the SF-36 – was excluded from analysis of that domain for that subject only.

## Results

### Patient characteristics

A total of 881 invitations were sent, 262 of those responding were assessed as ineligible to participate, and 231 had a colonoscopy, yielding a participation rate in colonoscopy of 37.3% [7]. The mean age of participants who had a colonoscopy was 62 years (SD = 5), with 48% (110/231) being females; 120 colonoscopy participants were drawn from the electoral roll arm (49% female) and 111 from the general practice arm, (46% female). The place of residence of study participants was classified according to the six geographic divisions of the Australian Capital Territory; that of participants drawn from the Electoral Roll, but not those drawn from General Practitioners, was found to correspond closely with the population distribution in the Australian Capital Territory [7].

### Colonoscopy results

Adenomatous and hyperplastic polyps were found in 104 (45%) subjects undergoing colonoscopy (Table 1). A total of 30 minor adverse events were reported, and 2 serious adverse events resulting in hospital admission, comprising (i) bradycardia and (ii) vasovagal episode.

**Table 1:** Clinical outcomes of colonoscopy in 231 participants

	N	%
Subjects in whom polyps were identified	104	45.0
Subjects with advanced adenoma	8	3.5
Subjects with adenomatous polyps	74	32.0
Subjects with hyperplastic polyps	53	22.9
Any adverse events	33	14.3

### Health assessment

The screening assessment (Visit 1) at which the baseline SF-36 scores were obtained was a mean of 30 days before colonoscopy (median 23, range, 2–332). Visit 3, at which the post-colonoscopy SF-36 scores were assessed, was a mean of 36 days after colonoscopy (median 32 days, range, 3–312). The mean time between Visits 1 and 3, and therefore the mean time between the two measures of self-rated wellbeing, was 68 days (median 56 days, range, 25–365; standard deviation 34 days). A total of 225 SF-36 questionnaires were recorded for both Visits. Of these, 6 subjects had incomplete data in one or more domains. One of these subjects had experienced an adverse event (vasovagal episode) and data was incomplete in the Bodily Pain scale only.

There were no significant differences between the SF-36 scores obtained before colonoscopy in our subjects and a general population sample of a similar age range *[Shadbolt B and Craft PS. Extending the use of the SF-36 in clinical practice. Health and Quality of Life Outcomes, Accepted for Publication]*.

The physical health of subjects, as measured by the Physical Functioning Scale, was not significantly different after colonoscopy (Table 2). Role Limitations due to Emotions and Vitality scores of participants were significantly higher following colonoscopy. There were no significant associations between changes in SF-36 scores and the finding of adenomatous polyps at colonoscopy, presence of adverse events, participants' rating of discomfort during the colonoscopy or overall rating of satisfaction with the procedure (data not shown).

**Table 2:** Raw SF-36 scores before and after colonoscopy

**SF-36 Scale**	Mean Score BEFORE	Mean Score AFTER	N	Difference of means (AFTER-BEFORE)	SD	P value
PF: physical functioning	80.2	78.6	225	-1.6	15.5	0.13
BP: bodily pain	75.6	77.3	224	+1.7	21.6	0.23
RP: role limitations due to physical condition	82.6	85.2	219	+2.5	33.5	0.27
GH: general health perceptions	73.3	72.7	223	-0.5	12.2	0.51
SF: social functioning	88.1	88.5	223	+0.4	18.9	0.76
VT: vitality	64.6	66.5	224	+1.9	14.2	0.04*
RE: role limitations due to emotions	88.6	92.9	222	+4.4	28.8	0.03*
MH: mental health	78.0	79.9	224	+1.9	12.8	0.03*

SD = standard deviation; P value associated with two-tailed paired t test (*P < 0.05)

**Figure 1:**
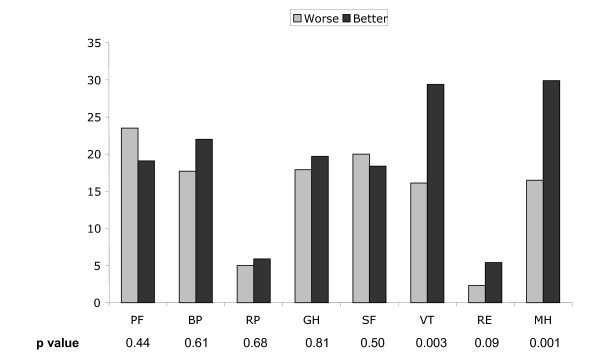
Percentage of CRC screening participants who had clinically worse (pale bars) or better (dark bars) SF-36 ranked scale scores after colonoscopy. P value was obtained from Wilcoxen Signed Ranks Test, using matched pairs of category comparisons before and after colonoscopy.

We next examined SF-36 rating scores according to predetermined categories. Most participants (range, 53.6% to 92.3%) were ranked in the same SF-36 category, signifying similar clinical status, before and after colonoscopy (Figure 1). Approximately 20% of participants overall had clinically worse scores (fall in category) after colonoscopy and 20% had clinically better (rise in category) scores. However, for the Role Limitations domain (Role Limitation due to Physical Condition and Role Limitation due to Emotions), more than 90% participants had similar scores before and after colonoscopy.

Considerably more participants (30% overall) had clinically better Vitality scores and Mental Health scores after colonoscopy (Figure 1). This finding was statistically significant (P < 0.05, Wilcoxon Signed-Rank Test).

We next examined relationships between clinical outcomes and QOL outcomes by category. There was no association between finding of adenomatous polyps at colonoscopy, presence of adverse events, or participants' overall rating of satisfaction with the procedure, and improvements or declines in category (data not shown).

## Discussion

Screening for cancer creates an increased level of duty for healthcare providers. Most patients having the screening test will not have the disease and will not benefit directly from the test. They may be harmed by a false-positive result or if the test causes injury. However, bowel cancer is an ideal disease for screening, with high lifetime incidence and prevalence in the screening age range [10], and being severe, detectable and curable with low morbidity at early stages of disease. Screening by colonoscopy is invasive and may cause haemorrhage in 1:500 patients and colonic perforation in 1:2000 [6] but has high specificity and low false-positive rate for cancer [10]. Because few will be harmed as a result of colonoscopic screening, any beneficial effects should be assessable. In contrast, any beneficial effects of other forms of colon cancer screening, such as faecal occult blood tests [11] might be obscured by the detrimental effect of a false-positive test, and the lack of confidence in a negative test. Therefore, we reasoned that quality-of-life assessment should be valid and meaningful after a single episode of colorectal cancer screening by colonoscopy.

In this study, asymptomatic, randomly selected participants having colonoscopic screening for colorectal cancer were surveyed for Health-related Quality of Life using the SF-36 before and after the screening colonoscopy. Aggregate data showed significant positive changes in SF-36 scores in the Mental Health, Role Limitations due to Emotions, and Vitality domains (Table 2). These measures do not indicate that the changes are clinically meaningful. Furthermore, numerical gains or losses from differing baselines are not necessarily equally weighted [12]. To further interpret these changes we examined SF-36 rating scores according to previously validated categories. These categories were constructed from an inpatient population with overall lower health status than our outpatient cohort, and were then validated in groups of patients with acute health conditions *[Shadbolt B and Craft PS. Extending the use of the SF-36 in clinical practice. Health and Quality of Life Outcomes, Accepted for Publication]*. Therefore, findings from Category analysis in the present study need to be interpreted with caution, but may serve to confirm and extend the results of aggregate data.

Nearly all participants had clinically similar Role Limitation Category scores (Figure 1) before and after colonoscopy, and while more participants showed significant gains than significant declines in Role Limitations due to Emotions, the number benefiting was small. Other domains exhibited more volatility, with approximately 40% of participants changing categories. The most conspicuous result was the 30% of participants reporting clinically meaningful gains in Vitality and Mental Health scores (Figure 1), confirming the results of raw-score analysis. Comparison of raw-score analysis and category analysis saw the improvement seen in Role Limitation due to Emotions (difference of means 4.4, P = 0.03 for two-tailed t-test) to be not statistically significant in Category analysis (P = 0.09).

Adenomatous polyps are rarely symptomatic. Therefore, even if subjects had a polypectomy at the screening examination, they could not be expected to improve in physical wellbeing. Indeed, in this study there were no improvements in the physical health domains of the QOL, irrespective of colonoscopy result. On the other hand, some subjects receiving good news might be expected to report improvements in mental health or emotional wellbeing. This was observed in the present study.

This is the first report of participants in a cancer screening program reporting an overall improvement in health ratings after the test. For example, men with a family history of prostate cancer were assessed before and after screening by prostate-specific antigen [5]. A third of these subjects reported a decline in more than 2 times the standard error in at least one dimension of the QOL scale. The mental component summary of the SF-36 scale exhibited an overall decline in participants in an ovarian cancer screening program [13]. In that study, participants were at increased risk of ovarian cancer and 38.5% of participants had a positive screening test.

Our study had some limitations. The second measure of QOL was generally performed within five weeks after the procedure, so the durability of any beneficial effect cannot be ascertained. We performed no measures of physical or mental functioning on participants, so we cannot determine whether the reported QOL gains were meaningful for these individuals. The results may have been subject to bias: the Authors are enthusiasts of colon cancer screening and may have unwittingly promoted colon cancer screening at interviews [14]. While the SF-36 scores obtained at the first visit did not differ from population norms, we cannot guarantee that they are 'baseline' for these individuals, and these initial scores may have reflected stresses induced by contact with an unfamiliar environment and medical staff. Therefore, any improvements may have reflected regression to the mean. Our results may only be relevant to our local population. The Australian Capital Territory has a relatively well-educated, high-income population [15] that may respond differently to other populations, although the SF-36 instrument itself is reported to be a robust tool across differing populations and languages [16].

## Conclusion

Average-risk persons participating in colon cancer screening with colonoscopy obtain a significant benefit, with improvements in Mental Health and Vitality domains of QOL. This improvement is not offset by declines in other domains of the QOL. If our observations can be reproduced in different populations, the improvement seen in quality of life after screening colonoscopy could represent a persuasive argument for screening for this type of cancer and using this modality. It would therefore be useful to confirm these findings in additional, prospective studies, performed in the average-risk population of different regions.

## Competing interests

The author(s) declare that they have no competing interests.

## Authors' contributions

DT conceived of the study, participated in its design and coordination, performed study procedures and helped to draft the manuscript. SC participated in design and coordination of the study and performed study procedures. MC participated in design and coordination of the study and performed study procedures. BS participated in the design of the study, performed the statistical analysis and helped to draft the manuscript. All authors read and approved the final manuscript.
